# Genomic Predictions in Korean Hanwoo Cows: A Comparative Analysis of Genomic BLUP and Bayesian Methods for Reproductive Traits

**DOI:** 10.3390/ani14010027

**Published:** 2023-12-20

**Authors:** Md Azizul Haque, Yun-Mi Lee, Jae-Jung Ha, Shil Jin, Byoungho Park, Nam-Young Kim, Jeong-Il Won, Jong-Joo Kim

**Affiliations:** 1 Department of Biotechnology, Yeungnam University, Gyeongsan 38541, Republic of Korea; azizul@ynu.ac.kr (M.A.H.); ymlee@yu.ac.kr (Y.-M.L.); 2 Gyeongbuk Livestock Research Institute, Yeongju 36052, Republic of Korea; hjjggo@korea.kr; 3 Hanwoo Research Institute, National Institute of Animal Science, Pyeongchang 25340, Republic of Korea; jins21@korea.kr (S.J.); bhpark70@korea.kr (B.P.); rat1121@korea.kr (N.-Y.K.)

**Keywords:** Bayesian methods, GBLUP, genomic prediction, Hanwoo, reproductive traits

## Abstract

**Simple Summary:**

Historically, Korea has been an agrarian society where cattle played a significant role in Korean culture as working animals. However, in recent decades, there has been an increase in meat demand and the expansion of the Korean economy. This shift has led to the predominant use of native Korean Hanwoo cattle for beef production since the 1960s. Genetic improvement programs for Hanwoo cattle traditionally focused on carcass and growth qualities due to the accessibility of trait information and the simplicity of analysis techniques, rather than reproductive traits. However, there has been a recent surge of interest in genetic analyses of the reproductive traits of Hanwoo cattle because these traits significantly impact calf productivity. To enhance beef production and the overall profitability of Hanwoo farming, it is imperative to implement genomic predictions for age at first calving (AFC), calving interval (CI), gestation length (GL), and number of artificial inseminations per conception (NAIPC) to better understand and improve their response to selection. This study aimed to estimate heritability and the accuracy of genomic estimated breeding values (GEBVs) using genomic best linear unbiased prediction (GBLUP) and Bayesian methods (BayesB, BayesLASSO, and BayesR) for traits under study. Our analysis revealed relatively lower heritability values for these traits and indicated that the accuracy of genomic prediction across all methods applied was similarly reduced, likely due to the inherent lower heritability of reproductive traits. As a result, the findings of this study provide valuable insights into the genetic breeding programs of the beef cattle industry.

**Abstract:**

This study aimed to predict the accuracy of genomic estimated breeding values (GEBVs) for reproductive traits in Hanwoo cows using the GBLUP, BayesB, BayesLASSO, and BayesR methods. Accuracy estimates of GEBVs for reproductive traits were derived through fivefold cross-validation, analyzing a dataset comprising 11,348 animals and employing an Illumina Bovine 50K SNP chip. GBLUP showed an accuracy of 0.26 for AFC, while BayesB, BayesLASSO, and BayesR demonstrated values of 0.28, 0.29, and 0.29, respectively. For CI, GBLUP attained an accuracy of 0.19, whereas BayesB, BayesLASSO, and BayesR scored 0.21, 0.24, and 0.25, respectively. The accuracy for GL was uniform across GBLUP, BayesB, and BayesR at 0.31, whereas BayesLASSO showed a slightly higher accuracy of 0.33. For NAIPC, GBLUP showed an accuracy of 0.24, while BayesB, BayesLASSO, and BayesR recorded 0.22, 0.27, and 0.30, respectively. The variation in genomic prediction accuracy among methods indicated Bayesian approaches slightly outperformed GBLUP. The findings suggest that Bayesian methods, notably BayesLASSO and BayesR, offer improved predictive capabilities for reproductive traits. Future research may explore more advanced genomic approaches to enhance predictive accuracy and genetic gains in Hanwoo cattle breeding programs.

## 1. Introduction

Genetic selection in the Korean beef sector has greatly improved commercially significant features. The most common beef breed on the Korean Peninsula is the locally bred Hanwoo cattle because of its distinctively flavored beef and superb flesh marbling. This unique breed of cattle has long been recognized as a potential genetic resource for enhancing production, with a history spanning a century. In ancient times, during the agricultural age, Hanwoo played a pivotal role as a draft animal and was occasionally involved in sacrificial rituals [1]. However, due to their limited population and the influence of religious and political factors in Korea, the utilization of Hanwoo cattle as a source of edible meat remained minimal. It was not until the 1960s, coinciding with the rapid growth of the Korean economy, that full-scale production of Hanwoo as a meat-type cattle breed commenced [2]. Consequently, the consumption of Hanwoo beef as a primary meat source in Korea has a relatively short history, despite the current scale of meat consumption in the country.

The genetic improvement efforts within the Hanwoo cattle-breeding program, owing to the accessibility of trait information and the simplicity of analytical techniques, primarily concentrated on enhancing carcass quality and growth characteristics. This emphasis stemmed from the substantial economic importance associated with these traits. However, a noteworthy shift has occurred in recent times as genetic analyses of reproductive traits in Hanwoo cattle have garnered considerable attention. Reproductive traits hold significant economic importance for sustainable food production, particularly in the case of monotocous livestock like cattle and buffalo. Enhancing fertility represents the best choice not only for reducing culling costs but also for conserving valuable genetic resources and increasing farm profitability. The challenge in improving reproductive efficiency has historically been due to factors such as low heritability, the binomial nature of a short-controlled breeding season, or the delayed expression of traits over the course of an animal’s life [3].

The productivity and, ultimately, the profitability of a cow–calf operation is influenced significantly by reproductive performance. A shift in the calving pattern brought on by a delay in conception because inadequate fertility lengthens the calving interval and increases the risk of culling. The primary goal of the cow breeding program is to enhance production rates, but this objective has occasionally resulted in unintended consequences such as reduced reproductive performance, including lower fertility and suboptimal embryo development [4,5]. The sooner a heifer gives birth, the more time she has to recover and start cycling again before the following breeding season.

Understanding the factors affecting fertility is a crucial indicator for management decisions on farms. Fertility is considered the most important attribute in beef cattle production from an economic point of view. Neglecting the reproductive features lowers the economic profit of a farm enterprise. Poor reproductive characteristics raise production costs by increasing the fertility treatment cost, the culling rate, the number of calving intervals, and the insemination number. Long-term production trait selection has resulted in a decline in reproductive performance among high-yielding breeds [6]. Age at first calving, calving interval, gestation length, number of conceptions per service, and various other indicators are used to assess the reproductive potential in cattle. A cow must be bred 80–85 days after calving to maintain an annual calving interval [7]. The interval between calving and conception directly affects the profitability of the beef sector [8] and the generation interval, which in turn controls the selection intensity. A heifer has more time to recover and restart cycling before the start of the following breeding season if she gives birth earlier in the calving season (first 21 days), which will help her maintain a 365-day calving interval [9]. The calving interval is the time period between the birth of one calf and the subsequent birth in the reproductive cycle of a cow. The gestation length has a crucial influence on breeding programs because it enables animals with longer gestations to require fewer days open, thereby reducing and enhancing the reproductive efficiency of cow herds while maintaining a 365-day calving interval.

The age at first calving (AFC) marks when a cow reaches sexual maturity and has offspring for the first time. In the cattle breeding system, the AFC is a complicated feature that includes puberty and the capacity to conceive, carry, and deliver a calf [10]. Early AFC is a reproductive feature that ensures profitable and sustainable beef production because of its relationship to annual earnings and rearing costs [11,12]. Body weight [13] and fat percentage [14] have a significant impact on the ability of a cow to reproduce.

Artificial insemination (AI), the main breeding technique for Korean Hanwoo cattle, has been used on more than 90% of Hanwoo cows in breeding programs [15]. On a farm, it is normally possible to produce one viable calf (ideally a heifer) from one cow in a calendar year (365 days) [16]. On the other hand, the first service following calving is often delayed and occasionally repeated because of the rapid increase in output. Increased services per conception are frequently signs of issues with the cow reproductive system, which harm farm economics [17] and frequently lead to herd culling [18]. The number of artificial inseminations per conception is influenced by several outside variables, including the composition and suitability of the ration for the cows’ physiological requirements, feeding frequently, housing arrangement of farms, the time of year and weather, the caliber of the veterinary staff’s work, and the proper observation of heat. Conception at the first service after calving is essential for improving the reproductive efficiency of cows and boosting profit. The non-return rate within 56 days of the initial insemination can be used to determine the success of insemination early on [19]. The ability of female cattle to conceive and maintain pregnancy throughout the early stages of gestation could be evaluated using this.

The effectiveness of genomic evaluations is significantly influenced by the statistical model used and the information available [20]. One of the earliest methods for determining breeding values is traditional pedigree-based BLUP (best linear unbiased prediction), which uses phenotypic records for a trait in an individual and information on their relatives [21]. Recently, the most popular strategy for genomic selection has relied on DNA-level information because it can accelerate genetic improvement more effectively than phenotypic data alone. Various statistical techniques have been applied to estimate the breeding values based on the genomic information (GEBV), with the BLUP and Bayesian approaches standing out as the most prominent. Genomic prediction through BLUP can be implemented via two equivalent models [22]. One approach involves the direct estimation of SNP effects, referred to as SNP-BLUP [23]. The other method involves the computation of a genomic relationship matrix from SNP genotypes, known as genomic BLUP (GBLUP) [22,24]. Alternative models posit that SNP effects follow non-normal distributions. For instance, the BayesA model assumes a Student’s t distribution for SNP effects [23], while BayesB [23], BayesC, BayesCπ [25], and BayesR [26] used mixture distributions, and BayesLASSO [27] uses exponential distributions. GBLUP methods with real data have demonstrated accuracy levels in genomic prediction that are akin to those achieved by non-normal distribution methods like BayesA, BayesB, and BayesR, particularly when moderate SNP densities are employed [28,29,30].

As noted by multiple authors, GBLUP offers the advantage of computational efficiency [31,32]. However, for traits influenced by quantitative trait loci (QTL) of moderate to large effect, Bayesian methods can yield higher prediction accuracies compared to GBLUP [33,34]. Furthermore, genomic prediction models that assume non-normal distributions of effects tend to achieve superior results to GBLUP when a substantial number of SNPs are employed, especially for multi-breed and across-breed predictions [26,35,36]. Nonetheless, this computational superiority comes at a cost, particularly when dealing with a large number of SNPs [37]. For instance, the BayesB method may result in the highest prediction accuracy in specific scenarios, but its computational time significantly increases with a large number of SNPs since it employs a Metropolis–Hastings algorithm [37].

On the other hand, methods like BayesA, BayesLASSO, and BayesR typically use Gibbs sampling, which, although faster than the Metropolis–Hastings algorithm, remains relatively sluggish when confronted with a large number of SNPs across numerous individuals [37]. BayesR shares some similarities with BayesB, allowing for SNP effects to be zero, moderate, or substantial [26]. However, it stands out as more computationally efficient, as it derives the proportion of SNPs in each normal distribution directly from the data, rather than relying on pre-set constants as in BayesB. Consequently, BayesR can approximate a wide spectrum of potential true distributions of SNP effects. When tested with actual data, BayesR attains prediction accuracies comparable to those of BayesA and BayesB [37]. While these Bayesian genomic prediction models are primarily employed for phenotypic prediction, they also offer valuable per-SNP information, including posterior estimates of effect size and variance, which could be instrumental for conducting QTL mapping [38].

While some research has been published based on genetic parameter estimations for reproductive traits [39,40], the accuracy of genomic prediction in the context of Hanwoo cattle remains an underexplored area, with no notable studies addressing reproductive traits. The scarcity of literature addressing the accuracy of genomic predictions for Hanwoo reproductive traits prompted the primary objective of our investigation. Specifically, we evaluated four distinct methodologies namely GBLUP, BayesB, BayesLASSO, and BayesR, which exhibit varying assumptions concerning the genetic architecture of these traits. Our primary objective was to compare these methods in terms of their cross-validation accuracies for predicting key reproductive traits such as age at first calving, calving interval, gestation length, and number of artificial inseminations per conception. This comprehensive investigation represents a significant step toward enhancing our understanding of the genomic prediction landscape within the Hanwoo cattle breed, shedding light on the suitability and effectiveness of various prediction methodologies for these crucial reproductive traits.

## 2. Materials and Methods

### 2.1. Ethics Statement

Genomic, pedigree, and phenotypic data associated with reproductive traits were collected in accordance with the protocol outlined by the Ministry of Agriculture, Food, and Rural Affairs, adhering to livestock laws in Korea. Hanwoo cows’ DNA was extracted from tail hair root samples collected by veterinarians. The pedigree information was recorded by the Korean Animal Improvement Association. Approval from the ethics committee was not necessary for this study, as all the phenotypic data were sourced from an existing database.

### 2.2. Animal Phenotypes

The first parity data were obtained from 11,348 Hanwoo cows from nine commercial herds in the South Korean province of Gyeongsanbuk-do. Pedigree data from 27,172 individuals were used in the animal model. The age at first calving (AFC), calving interval (CI), gestation length (GL), and the number of artificial inseminations per conception (NAIPC) were the four female reproductive traits that were examined. The AFC, CI, and GL were measured in days, while NAIPC was recorded as the total number of records. Table 1 represents the summary statistics for the reproductive traits to estimate the variance components and the estimation of breeding values.

### 2.3. Genotyping and Quality Control

A total of 11,348 Hanwoo cows were genotyped using an Illumina Bovine 50K SNP chip (Illumina Inc., San Diego, CA, USA), where 53,866 SNPs were embedded. To ensure data quality, we first removed SNPs located on sex chromosomes in duplicate or uncertain positions, resulting in the elimination of 1750 SNPs, leaving us with 52,116 SNPs for analysis. Additionally, we excluded 4 animals with discrepancies between pedigree and genomic relationships from further analyses. For the subsequent analysis, we implemented multiple quality control (QC) criteria to filter out low-quality SNPs. Specifically, SNPs with a minor allele frequency (MAF) of less than 5% (i.e., monomorphic; 9281 SNPs), a SNP call rate below 90% (732 SNPs), individuals with a genotyping call rate less than 90% (N = 58), and SNPs showing a significant deviation from Hardy–Weinberg Equilibrium (HWE) with a *p*-value greater than 10^−6^ (1296 SNPs) were excluded from the dataset. The identity-by-state (IBS) test was also performed to determine if the datasets had genetically similar individuals or genotyping errors. The pair of individuals showing a similarity rate >99% indicates an identical animal or error in genotyping (N = 48). The IBS and entire QC process were performed using the PLINK v1.9 toolset [41]. After IBS and QC, finally, 11,238 animals with genotypes of 40,807 SNPs were available for further analysis.

### 2.4. Statistical Analysis

#### 2.4.1. Estimation of Variance Components

The variance components and heritabilities for each reproductive trait were estimated using the restricted maximum likelihood method (REML) for animal models using BLUPF90+ v2.52 software [42]. A single-trait pedigree-based animal model is as follows:

(1) y=Xb+Zu+e

where y is the vector of phenotypes; b is the vector of the fixed effects, including the herd in which the animal was raised, and the year and season of birth and calving; u is the vector of additive genetic effects of the individuals; X is the incidence matrix of b; Z is the incidence matrix of u; and e is the vector of the residuals. It was assumed that $u\sim N(0,A\sigma_{a}^{2}$ and $e\sim N(0,I\sigma_{e}^{2})$, where A is the pedigree-based genetic relationship matrix and $\sigma_{a}^{2}$ is the additive genetic variance, and $\sigma_{e}^{2}$ is the residual variance. The adjusted phenotypes $y_{c}$ were obtained for each trait and animal as the residual effects of the y=Xb+e, which $\hat{b}={\left(X^{\prime }X\right)}^{-1}X^{\prime }y$.

#### 2.4.2. Estimation of Breeding Values

Genomic predictions were performed for animals with both genotype and phenotypic records (Table 1) using four different statistical models: GBLUP [22,24], BayesB [23], BayesLASSO [27], and BayesR [26].

#### Genomic Best Linear Unbiased Prediction Model (GBLUP)

The GBLUP model was implemented using ASReml-SA v4.2 software [43] as follows:

(2) $$y_{c}=1\mu +Zg+e$$

where $y_{c}$ is the vector of adjusted phenotypes; 1 is the vector of ones; μ is the overall mean; Z is the incidence matrix of g; e is the vector of residuals; and g is the additive genetic effects of individuals with var($g_{i})=G\sigma_{a}^{2}$, where G is the genomic relationship matrix (GRM) constructed using SNP information as follows [22]:

(3) $$G=\frac{{MM}^{\prime }}{2\sum_{i=1}^{n}p_{i}(1-p_{i})}$$

where n is the total number of markers (40,807); $p_{i}$ is the allele frequency of the ith marker; and M is the matrix of centered genotypes. The genomic relationship matrix (GRM) was constructed using the genome-wide complex trait analysis (GCTA) tools [44], which effectively retain the genomic relationship between animals [22].

#### Bayesian Model

The statistical model of genomic prediction was performed under the BayesB, BayesLASSO, and BayesR using the following model [25]:

(4) $$y_{c}=1\mu +\sum_{i=1}^{m}z_{i}g_{i}+e$$

where $y_{c}$ is the vector of adjusted phenotypes; 1 is the vector of ones; μ is the overall mean; m is the number of SNPs; $z_{i}$ is the vector of genotypes of fitted marker i; $g_{i}$ is the additive effect of that SNPs; and e is the vector of residual effects.

In the BayesB method, the prior distribution of the effect of each marker is a mixture of scaled-t distribution with probability π and a distribution of point mass at zero with probability (1 − π). Based on this assumption, a prior distribution of the variances of the effects on the markers can be written as:
$\sigma_{gi}^{2}=0$with probability π$\sigma_{gi}^{2}\sim \chi^{-2}(v,S)$with probability (1 − π) where π is the proportion of markers with null genetic effects. The BayesB analysis was performed using the GenSel v4.90 program [45]. A total of 41,000 iterations of Markov chain Monte Carlo (MCMC) were run for the analysis after discarding the first 1000 iterations of the burn-in period and each of the 100 iterations was selected to calculate posterior mean and variance for the marker effects.

In the BayesLASSO model, prior densities of the non-zero SNP effects are assumed to be double exponential, and also, the marker effects have locus-specific variance. The prior distribution for $g_{i}$ follows a normal distribution $N(0,I\sigma_{\alpha }^{2})$ and the prior distribution is implemented as follows [46]:

(5) $$\mathrm{Pr}\left(g_{i}|\tau^{2}\right)=N(0,\tau_{i}^{2})$$

(6) $$\mathrm{Pr}\left(\tau_{i}^{2}\right)=\frac{\lambda^{2}}{2}exp(-\lambda^{2}\left|\tau_{i}^{2}\right|)$$

The prior distribution for $\sigma_{g}^{2}$ was an inverted $\chi^{2}$ distribution with two degrees of freedom and expectations equal to the value used in regular genetic evaluation as $\sigma_{g}^{2}/(1-\pi )\sum_{i=1}^{m}{2p}_{i}q_{i}$ [25]. The BayesLASSO analysis was performed using the software GS3 [47] (available at http://snp.toulouse.inra.fr/~alegarra/).

In the BayesR model, marker effects are sampled from a mixture of four normal distributions with mean zero and variances equal to 0, 0.0001 $\sigma_{g}^{2}$, 0.001 $\sigma_{g}^{2}$, and 0.01 $\sigma_{g}^{2}$:

(7) $$\beta_{i}\;\sim \;\pi_{1}N\left(0,0\;\sigma_{g}^{2}\right)+\pi_{2}N\left(0,0{.0001\sigma }_{g}^{2}\right)+\pi_{3}N\left(0,0.001\;\sigma_{g}^{2}\right)+\pi_{4}N\left(0,0.01\;\sigma_{g}^{2}\right)$$

where $\sigma_{g}^{2}$ is the additive genetic variance for each trait. Marker effects were estimated using the Markov chain Monte Carlo (MCMC) approach and software implemented in BayesR [48] with default settings.

#### 2.4.3. Cross Validation

We adopted a repeated fivefold cross-validation (CV) approach to measure genomic prediction accuracy. To elaborate, the experiment underwent 100 repetitions, with a 5-fold CV procedure employed in each iteration [49,50,51]. Consequently, a training–testing protocol replicated five times was used. As described by Badke et al. [52], this method is referred to as the cross-validation technique, designed to minimize sampling errors. The entire population was divided randomly into 5 equal groups for this 5-fold cross-validation procedure. Thus, one division of data (20%) serves as a validation or testing group and the other four divisions (80%) turn into the reference or training group. This process was repeated 5 times to provide each animal in the dataset a chance to be included in the testing and reference groups.

#### 2.4.4. Accuracy of Genomic Prediction

The prediction accuracy was evaluated using Pearson’s correlation coefficient (r) between the adjusted phenotypes of the individuals in the validation dataset and their GEBV divided by the square root of the heritability for each trait [53]. The accuracy for each replicate was obtained as the mean of the accuracies for the fivefold cross-validations of the 100 replicates [51]. The empirical standard error (SE) was determined by dividing the standard deviation of the five calculated accuracies from the fivefold cross-validation (CV) by the square root of 5. Additionally, the slope of the regression of phenotype on GEBV was computed to measure the bias in the GEBV [51]. A regression coefficient close to 1 indicates no bias, while a slope of <1 or >1 suggests the underestimation or overestimation of GEBV, respectively [54,55].

#### 2.4.5. Estimation of Genomic Heritability

The genomic heritability ($h_{g}^{2}$) was calculated as follows [56]:

(8) $$h_{g}^{2}=h^{2}\frac{\sigma_{g}^{2}}{\sigma_{a}^{2}}$$

where $h^{2}$ is the pedigree-based heritability and $\sigma_{a}^{2}$ is the pedigree-based additive genetic variance. On the other hand, $\sigma_{g}^{2}$ is the genetic variance obtained from GBLUP, BayesB, BayesLASSO, and BayesR, respectively.

## 3. Results and Discussion

### 3.1. Description of SNP Statistics

**Figure 1:**
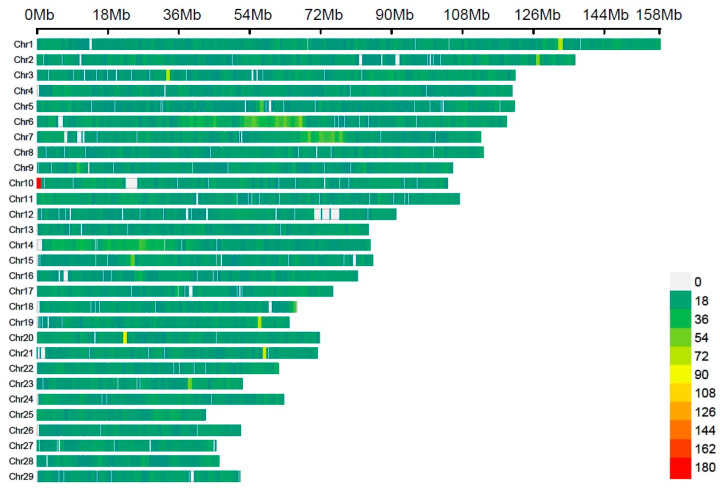
SNP density plot for each chromosome displaying the quantity of SNPs.

After undergoing quality control procedures, which encompassed 78.30% of the initial SNPs on all 29 *Bos taurus* autosomes (BTA), a selection of 40,807 common SNPs was made. The distribution of these markers was uneven, with a significant overrepresentation of specific chromosomes (as shown in Table S1 and Figure 1). Among these, BTA1 had the highest number of SNPs (2570), covering approximately 158 Mb, while BTA28 had the fewest SNPs (705), spanning about 46.1 Mb. Additionally, BTA1, BTA2, BTA3, and BTA6 each had more than 2000 SNPs.

### 3.2. Genomic Relationship Matrix

**Figure 2:**
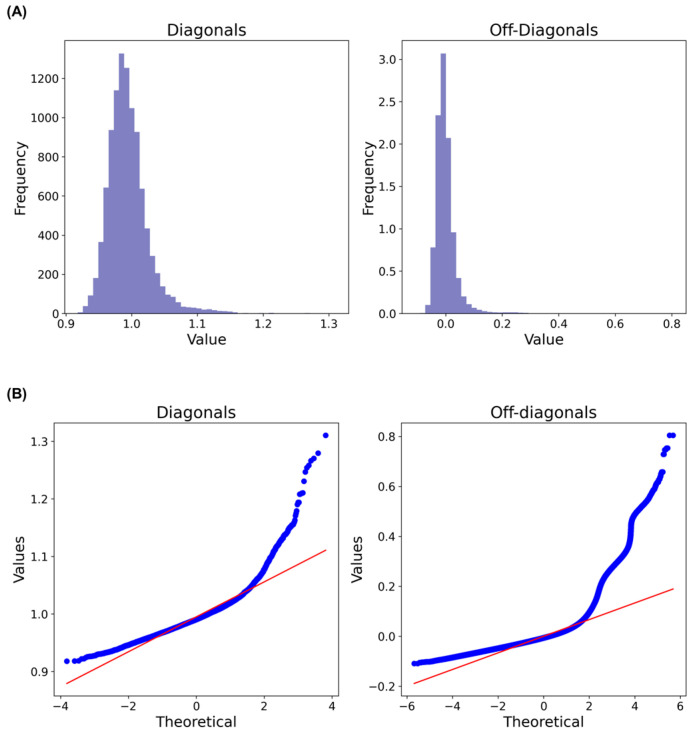
(**A**) Histograms and (**B**) QQ plots of the diagonal and off-diagonal values of the genomic relationship matrix.

The GRM for Hanwoo cows was calculated using the method proposed by VanRaden [22]. Analysis of the distribution of both the diagonal and off-diagonal values of the GRM (Figure 2A) did not reveal multiple distinct peaks. However, it is worth noting that the distributions were not perfectly normal, as demonstrated by the quantile–quantile (QQ) plots of the diagonal and off-diagonal values of the GRM (Figure 2B). Nevertheless, a clear genotype cluster was observed in the dataset (Figure S1). Additionally, when examining the plot of the first three principal components (PCs), it was found that they explained 24.23%, 22.16%, and 19.83% of the variations, respectively (Figure S1).

### 3.3. Estimation of Heritability

Pedigree-based estimates of variance components and heritability (h^2^) for reproductive traits are provided in Table 2. In our comprehensive analysis of h^2^ estimates for Hanwoo cows’ reproductive traits, we explored both pedigree-based h^2^ and genomic h^2^ using various approaches. The pedigree-based h^2^ assessments revealed consistently low h^2^ values across all studied traits. Specifically, the AFC showed a h^2^ of 0.070, while the CI demonstrated a h^2^ of 0.026. Moreover, GL exhibited a h^2^ estimate of 0.102, and NAIPC displayed the h^2^ at 0.055. Among the reproductive traits, GL had the highest estimated h^2^ value.

Expanding our investigation to include genomic methodologies such as GBLUP, BayesB, BayesLASSO, and BayesR, the estimations varied noticeably from the pedigree-based assessments (Table 3). GBLUP showed a marked decrease in h^2^ estimates compared to PBLUP for all traits. For instance, AFC was estimated at 0.039, GL at 0.061, CI at 0.022, and NAIPC at 0.030, representing a reduction in h^2^ compared to the pedigree-based method. Bayesian methods (BayesB, BayesLASSO, and BayesR) also provided genomic h^2^ estimates for the studied traits. These estimates varied from the PBLUP and GBLUP results. Notably, these methods generally produced estimates that were closer to GBLUP values than the higher estimates from the PBLUP approach. For example, AFC h^2^ estimates ranged between 0.039 and 0.041 for Bayesian methods, while estimates for CI, GL, and NAIPC were in the range of 0.022 to 0.024.

The comparison across the methods and traits reveals the consistent trend of higher h^2^ values estimated by PBLUP in contrast to genomic methods. While there were variations among Bayesian methods, they generally aligned more closely with GBLUP estimates. Additionally, the difference between PBLUP and GBLUP was more prominent for certain traits such as AFC, indicating a significant disparity in the h^2^ assessments derived from pedigree and genomic-based methods. GL estimates exhibited a significant difference, showing reduced h^2^ in all genomic methods in comparison to PBLUP. NAIPC estimates displayed intermediary values between PBLUP and GBLUP in the Bayesian models.

Among the reproductive traits, the GL exhibited the highest estimated h^2^ values in both pedigree-based and genomic methodologies. These consistently low h^2^ estimates align with prior research findings. Lopez et al. [40] reported similarly low h^2^ estimates for CI, GL, and AFC in Hanwoo cattle, specifically 0.01, 0.14, and 0.08, respectively. Notably, h^2^ values of 0.049 [57] and 0.047 [58] for CI, 0.215 [58] and 0.158 [57] for AFC, and 0.020 [57] for NAIPC have been described for Japanese Black (Wagyu) cattle. Yague et al. [59] reported h^2^ estimates of 0.085, 0.037, and 0.071 for CI, GL, and NAIPC, respectively. In comparison to other breeds, CI h^2^ estimates reached 0.222 in Jersey ⅹ Red Sindhi cattle [60], 0.105 [61], and 0.02 [62] in Nelore cattle, and 0.09 in Brahman–Angus cattle [63]. Adonai Alejandro et al. [64] noted a notably high h^2^ value for AFC in Simmental cattle.

The relatively low genomic h^2^ observed for reproductive traits suggests the need for a larger number of animals with both genotypes and phenotypes to accurately estimate SNP effects. The 50K SNP chip proves insufficient in capturing all genetic variability for these traits, prompting consideration of a high-density SNP chip for better assessment of linkage disequilibrium (LD) and potentially capturing a significant portion of the additive genetic variance [65]. Additionally, PBLUP relies on pedigree information, which may be limited in accurately capturing genetic relationships, especially in cases of incomplete or inaccurate pedigrees. Genomic evaluation, in contrast, utilizes information directly from genetic markers, offering a more precise representation of genetic relatedness [66]. Studies in livestock genomics have indicated that SNP chips explain only a relatively small proportion of h^2^ [67,68].

The variations in h^2^ can be attributed to breed-specific genetic differences and genotype-environment interactions. These variations in h^2^ are influenced by genetic makeup and environmental differences, which can vary from one herd to another or even within the same herd from year to year [69]. Reproductive traits are inherently complex and subject to substantial environmental variability, including nutritional, climatic, and management influences. The underlying genetic architecture of these traits likely involves various genes with small effects, which further complicates h^2^ estimation. This multifactorial nature of reproductive traits underscores the challenge of accurately estimating higher h^2^, resulting in the observed lower heritability values in beef cattle reproductive traits. A shift in any of the factors influencing h^2^ can result in an increase or decrease in its estimate. An increase in h^2^ estimates arises from higher genetic variance or reduced environmental variance, while a decrease arises from elevated environmental variance or reduced genetic variance [70].

The low h^2^ values in reproductive traits are largely attributed to the substantial influence of non-genetic effects on the variability of reproductive parameters, rendering genetic improvements in these traits and overall cattle production quite challenging [71]. As substantiated by this study and the research of other authors, reproductive traits exhibit low hereditary properties and are primarily governed only by weak additive genetic effects.

### 3.4. Evaluation of GEBV Prediction Accuracy

**Figure 3:**
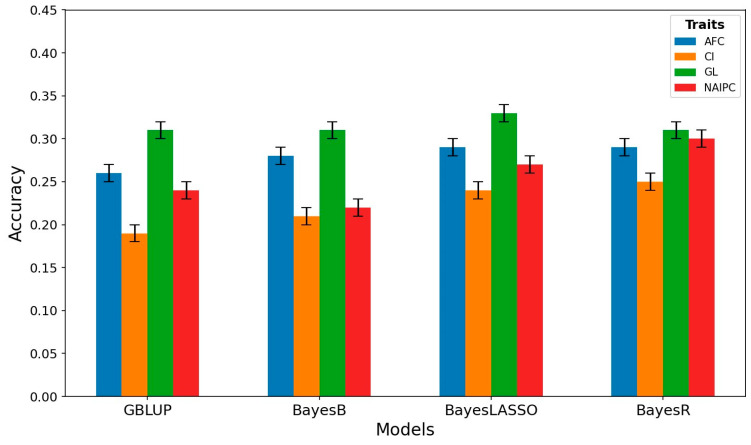
Accuracy of genomic predictions obtained by different methods in Hanwoo cows’ reproductive traits. Vertical lines indicate the empirical standard error for cross-validation results.

In this study, we assessed the genomic prediction accuracy for reproductive traits in Hanwoo cows using GBLUP, BayesB, BayesLASSO, and BayesR (Figure 3). In the case of AFC, GBLUP demonstrated an accuracy of 0.26, while BayesB, BayesLASSO, and BayesR displayed slightly higher accuracies of 0.28, 0.29, and 0.29, respectively. For CI, GBLUP achieved an accuracy of 0.19, whereas BayesB, BayesLASSO, and BayesR performed comparatively better, with accuracies of 0.21, 0.24, and 0.25, respectively. In terms of GL, GBLUP, BayesB, and BayesR exhibited equal accuracies at 0.31, while BayesLASSO showed a slightly superior accuracy of 0.33. For NAIPC, GBLUP achieved an accuracy of 0.24, while BayesB, BayesLASSO, and BayesR displayed accuracies of 0.22, 0.27, and 0.30, respectively.

The transition from GBLUP to Bayesian methods for AFC indicated marginal increases in accuracy. Although the Bayesian methods showed slightly higher accuracies, the differences were relatively subtle, ranging from 0.02 to 0.03 increases compared to GBLUP. AFC predictions using these methods showed a modest advantage but did not substantially outperform GBLUP. However, focusing on CI revealed more noticeable improvements in accuracy when transitioning from GBLUP to Bayesian methods. The Bayesian methods consistently exhibited higher accuracies compared to GBLUP, with increases ranging from 0.02 to 0.06, demonstrating a substantial advantage of Bayesian methods in estimating CI compared to GBLUP. In the case of GL, the GBLUP, BayesB, and BayesR displayed equivalent accuracies, while BayesLASSO demonstrated a slightly higher accuracy. The increase in accuracy for BayesLASSO was 0.02, indicating its slightly enhanced performance in estimating GL compared to the other methods. Despite the marginal increase, it is notable that BayesLASSO showed a modest advantage in accuracy for predicting GL. For NAIPC, the Bayesian methods showed substantial increases in accuracy when compared to GBLUP. Transitioning to BayesLASSO and BayesR from GBLUP led to increases in accuracy ranging from 0.03 to 0.06. These increases signify a significant advantage of the Bayesian methods in predicting NAIPC accurately compared to GBLUP.

The comparison of results demonstrates that Bayesian methods, especially BayesLASSO and BayesR, consistently outperformed GBLUP in predicting CI and NAIPC, showcasing higher increases in accuracy for these traits. The AFC predictions using Bayesian methods showed slight advantages but not as prominently as for CI and NAIPC. Additionally, GL predictions did not display a notable variance in accuracy among the methods, although BayesLASSO showcased a slight edge. The superior accuracies observed in Bayesian methods for CI and NAIPC might be explained by their enhanced capacity to effectively capture the genetic factors that contribute to these traits. Bayesian methods excel in handling complex genetic architectures by considering various levels of effects for individual markers. This flexibility allows them to identify and account for specific genetic variants with varying contributions to the traits. In contrast, GBLUP, while widely used, may oversimplify the genetic architecture by assuming a constant effect for all markers, potentially missing out on crucial information embedded in the genetic variations. Therefore, the Bayesian methods, with their adaptability to diverse genetic scenarios, showcase improved predictive performance, providing more accurate estimates for CI and NAIPC [72].

The accuracy of genomic evaluation for reproductive traits is significantly influenced by various factors such as breeds, the genetic architecture of the studied traits, the statistical approach, the effects of SNPs, and the specific SNP set used. Our study investigating the genomic prediction accuracy for reproductive traits in Hanwoo cows uncovered varying degrees of precision across different methodologies. Notably, our study revealed distinct accuracy values for each trait when using GBLUP, BayesB, BayesLASSO, and BayesR methods. For example, in our findings, the accuracy of GBLUP for the AFC was 0.26. This was in comparison to previously reported accuracies by Laodim et al. [73] for Thai crossbreed animals, which yielded a figure of 0.299. Additionally, accuracy values for AFC in Nelore cattle ranged from 0.23 to 0.33, as per three Bayesian statistical approaches [74]. These findings roughly correlate with our present findings. In other studies, the average prediction accuracy for these traits in Nelore cattle ranged from 0.38 to 0.42 using GBLUP and Bayesian methods [72], which exceeded the accuracies observed in our study. Moreover, when applying the BayesC technique to Nelore cattle, Boddhireddy et al. [75] reported a much higher prediction accuracy of 0.64 compared to our current findings.

The current findings are in line with previous research that highlighted the superiority of Bayesian methods over GBLUP in reproductive traits [72]. Luan et al. [76], in their analysis of productive and reproductive traits in Norwegian Red cattle, concluded that the choice of the method for estimating marker effects, and consequently GEBV, significantly relies on the specific trait under investigation. Their study revealed that GBLUP was slightly more accurate in estimating GEBV for production traits. However, for reproductive traits, the Bayesian models displayed superior performance, similar to the observations in this study. Bayesian methods in marker selection offer a more realistic assumption of a trait’s genetic architecture [46,77]. In this study, as only reproductive traits were examined, a comparable genetic architecture of these traits was anticipated. The superiority of Bayesian models over GBLUP across all traits might be due to a simpler genetic architecture, potentially influenced by a few loci exerting major effects [54].

Bayesian approaches would improve prediction accuracy, particularly with multi-trait analysis [78]. Although this study predicted the single-trait model, the prediction accuracy was also higher than that of the GBLUP model, indicating that Bayesian techniques performed better than GBLUP regarding reproductive traits. On the other hand, all phenotyped animals must have their genotypes considered for the genomic assessment to use Bayesian models [23]. In addition, modeling the distribution of marker effects is a benefit of Bayesian models over frequentist inference [23].

According to Meher et al. [51], the performance of Bayesian methods is better for traits regulated by a few QTLs/genes with relatively substantial effects. The GBLUP, however, showed greater genomic prediction accuracy for the traits controlled by several small-effect QTLs. Furthermore, Bayesian approaches outperformed GBLUP methods for highly heritable traits, but they were on par with traits that were not highly heritable [79]. Furthermore, according to Mehrban [65], Bayesian approaches are effective and more accurate.

### 3.5. Bias in Genomic Prediction Accuracy

**Figure 4:**
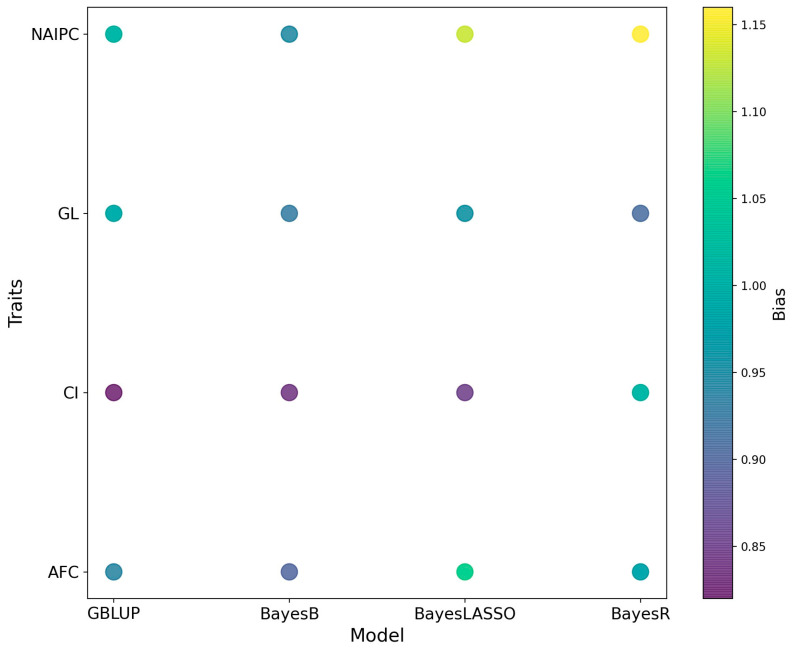
The bias estimation in genomic predictions for Hanwoo reproductive traits.

The bias in genomic predictions for all reproductive traits is shown in Figure 4. When comparing biases among different models for each trait, notable variations emerge. In the context of AFC, the GBLUP model showed a bias of 0.94, and BayesB followed closely with a bias of 0.90. However, BayesLASSO presented a notably higher bias at 1.06, and BayesR also exhibited a slightly increased bias of 0.98. For CI, GBLUP demonstrated a bias of 0.82, and BayesB had a slightly lower bias at 0.84. Meanwhile, BayesLASSO resulted in a bias of 0.85, and BayesR showed a similar bias at 1.01, highlighting a mild increase. When assessing GL, GBLUP stood at 0.99, closely resembling the biases of BayesB and BayesR with values of 0.93 and 0.91, respectively. BayesLASSO had a slightly increased bias at 0.96. For NAIPC, the biases were more varied. GBLUP reported a bias of 1.01, while BayesB had a slightly lower bias at 0.95. BayesLASSO presented a higher bias of 1.13, and BayesR showed the most increased bias of 1.16.

## 4. Conclusions

Our study aimed to assess the heritability and genomic prediction accuracy for age at first calving, calving interval, gestation length, and number of artificial inseminations per conception using various statistical methods. The findings revealed relatively low heritability values for these reproductive traits, emphasizing the challenges in predicting them accurately. Both pedigree-based and genomic heritability estimates exhibited limitations, indicating the inherent complexity of these traits’ genetic architecture. Genomic prediction accuracy varied among methods, with Bayesian approaches demonstrating slightly better performance compared to GBLUP. These results suggest that Bayesian methods, particularly BayesLASSO and BayesR, offer superior predictive capabilities for reproductive traits, specifically CI and NAIPC, due to their improved capacity to capture the underlying genetic factors influencing these traits. Furthermore, this phenomenon is particularly notable for reproductive traits influenced by few QTLs, with each having a larger effect on the genotypic variability. While AFC and GL predictions displayed more modest advantages with Bayesian methods, the findings highlight the potential for improved genetic selection strategies in the Hanwoo cattle industry, emphasizing the importance of considering reproductive traits in breeding programs. In terms of the bias in the accuracy of genomic predictions, BayesB displayed the least bias, whereas BayesR showed the highest bias among all the models considered. These biases underscore the significance of carefully selecting the prediction model in genomic studies, as it significantly impacts the accuracy of predictions for reproductive traits. Despite the advantages observed with Bayesian methods, accurately predicting reproductive traits in Hanwoo cattle remains a complex challenge, encouraging further research and the integration of more advanced methodologies for significant improvements. Acknowledging the limitations associated with the inherently low heritability of these traits is crucial, as it poses challenges in achieving substantial improvements. Future research may explore more advanced genomic approaches to further enhance predictive accuracy and genetic gains in Hanwoo cattle breeding programs.

## Figures and Tables

**Table 1:** Summary statistics for the Hanwoo reproductive traits.

Methods	Traits	Number of Animals	Mean	SD	Minimum	Maximum
Pedigree-based analysis	AFC (days)	11,348	748.74	85.75	447	1060
	CI (days)	8878	376.41	51.94	242	600
	GL (days)	11,348	287.19	8.20	206	395
	NAIPC (1–4)	11,348	1.45	0.81	1	7
Genome-based analysis	AFC (days)	10,148	736.18	64.43	556	934
	CI (days)	7994	370.56	40.04	300	497
	GL (days)	10,426	286.62	4.91	275	303
	NAIPC (1–4)	11,204	1.44	0.79	1	4

SD, standard deviation; AFC, age at first calving; CI, calving interval; GL, gestation length; NAIPC, number of artificial inseminations per conception.

**Table 2:** Estimated variance components and heritability for reproductive traits of Hanwoo cows using pedigree and phenotypic records.

Traits	$h^{2}$ ± SE	$\sigma_{a}^{2}$	$\sigma_{e}^{2}$	$\sigma_{p}^{2}$
AFC	0.070 ± 0.02	284.06	3756.37	4040.43
CI	0.026 ± 0.04	42.35	1612.31	1654.66
GL	0.102 ± 0.02	2.15	18.83	20.98
NAIPC	0.055 ± 0.01	0.03	0.52	0.55

SE, standard error; $h^{2}$, heritability; $\sigma_{a}^{2}$, additive genetic variance; $\sigma_{e}^{2}$, residual variance; $\sigma_{p}^{2}$, phenotypic variance; AFC, age at first calving; CI, calving interval; GL, gestation length; NAIPC, number of artificial inseminations per conception.

**Table 3:** Genomic variance, marker variance explained, and genomic heritability of Hanwoo cows’ reproductive traits.

Method	Traits	$\sigma_{g}^{2}$	$\frac{\sigma_{g}^{2}}{\sigma_{a}^{2}}$	$h_{g}^{2}=h^{2}\frac{\sigma_{g}^{2}}{\sigma_{a}^{2}}$
GBLUP	AFC	156.09	0.55	0.039
	CI	35.95	0.85	0.022
	GL	1.29	0.60	0.061
	NAIPC	0.02	0.56	0.030
BayesB	AFC	161.09	0.57	0.040
	CI	37.00	0.87	0.022
	GL	1.32	0.61	0.063
	NAIPC	0.01	0.43	0.024
BayesLASSO	AFC	159.28	0.56	0.039
	CI	39.71	0.94	0.024
	GL	1.23	0.57	0.059
	NAIPC	0.02	0.57	0.031
BayesR	AFC	167.33	0.59	0.041
	CI	39.73	0.94	0.020
	GL	1.29	0.60	0.061
	NAIPC	0.01	0.47	0.026

$\sigma_{g}^{2}$, genomic variance; $\frac{\sigma_{g}^{2}}{\sigma_{a}^{2}}$, marker variance explained; $h_{g}^{2},$ genomic heritability; $h^{2}$, pedigree-based heritability; AFC, age at first calving; CI, calving interval; GL, gestation length; NAIPC, number of artificial inseminations per conception; GBLUP, genomic best linear unbiased prediction.

## Data Availability

The data presented in this study are available on request from the corresponding author. The data are not publicly available due to privacy and confidentiality.

## Supplementary Materials

The following supporting information can be downloaded at: https://www.mdpi.com/article/10.3390/ani14010027/s1, Figure S1: Scatter plot of the first three principal components of the Hanwoo cows genotypic data; Table S1: SNP statistics after QC for Hanwoo cows autosomes.
