# Supplementary material for: Genomic Predictions in Korean Hanwoo Cows: A Comparative Analysis of Genomic BLUP and Bayesian Methods for Reproductive Traits
Source: Animals (Basel). 2023 Dec 20;14(1):27. doi: 10.3390/ani14010027 (PMC10778388; doi:10.3390/ani14010027)
Supplement: Supplementary file 1 [file animals-14-00027-s001.zip › animals-2726520-supplementary.pdf]

Table S1. SNP statistics after QC for Hanwoo cows' autosomes.

BTA	SNP before QC	SNP after QC	Removed frequency	Average distance (kb)	Standard deviation of distance (kp)	Minimum distance (kb)	Maximum distance (kb)
1	3221	2570	0.202	61.500	56.200	0.045	936.230
2	2756	2136	0.225	64.000	71.900	0.075	1402.860
3	2579	2003	0.223	60.500	63.700	0.011	863.150
4	2477	1891	0.237	63.500	54.900	0.026	623.660
5	2154	1650	0.234	73.400	74.000	0.036	988.850
6	3157	2506	0.206	47.500	57.100	0.033	1601.810
7	2478	1994	0.195	56.400	70.600	0.133	1518.760
8	2243	1749	0.220	64.700	55.400	0.084	547.230
9	2073	1593	0.232	66.200	64.300	0.449	642.760
10	2355	1846	0.216	56.500	93.300	0.070	3259.340
11	2179	1693	0.223	63.300	60.200	0.133	833.190
12	1650	1227	0.256	74.100	126.900	0.237	2470.220
13	1681	1302	0.225	64.400	56.100	0.382	715.700
14	2266	1786	0.212	46.600	46.900	0.005	505.770
15	1665	1290	0.225	65.700	66.600	0.010	969.410
16	1598	1220	0.237	66.800	71.600	0.178	1360.520
17	1567	1204	0.232	62.200	66.700	0.164	1301.140
18	1301	1030	0.208	63.300	65.900	0.513	1106.250
19	1377	1104	0.198	57.600	54.500	0.729	586.980
20	1568	1221	0.221	58.700	52.000	0.466	559.100
21	1397	1129	0.192	63.000	71.700	0.485	1322.350
22	1209	959	0.207	63.900	54.700	0.087	562.800
23	1124	904	0.196	57.700	57.000	0.321	488.530
24	1229	985	0.199	63.100	53.500	0.063	454.800
25	937	772	0.176	55.300	45.600	0.069	332.620
26	1030	806	0.217	63.300	50.300	0.281	394.540
27	917	736	0.197	61.700	62.000	0.151	587.190
28	902	705	0.218	65.600	57.900	0.023	555.530
29	1026	796	0.224	64.300	67.000	0.031	1060.190
**Total**	**52116**	**40807**	**0.216**	**61.890**	**63.741**	**0.182**	**984.534**

BTA, *Bos taurus* autosomes; QC, quality control.

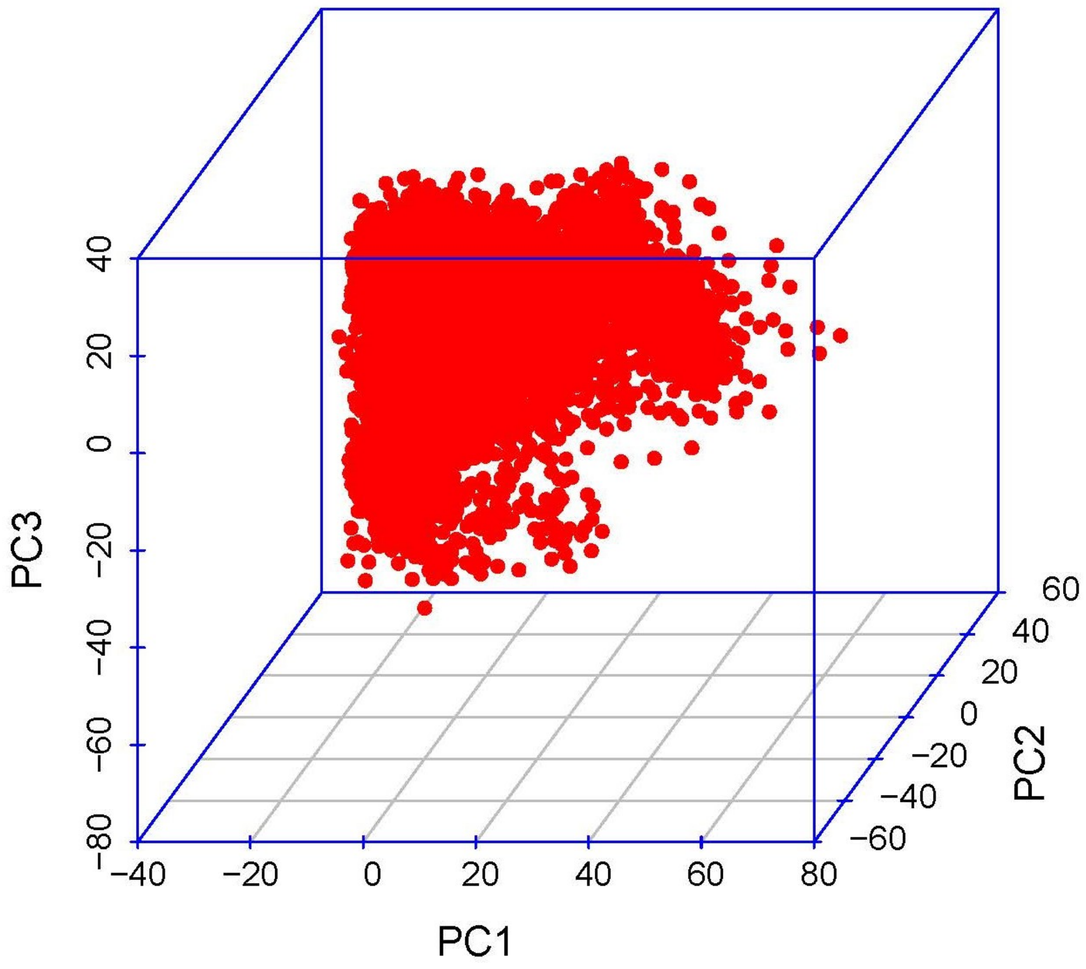

Figure S1. Scatter plot of the first three principal components of the Hanwoo cows' genotypic data.
